# Weight-adjusted dosing of tinzaparin for thromboprophylaxis in obese medical patients

**DOI:** 10.1016/j.rpth.2023.100054

**Published:** 2023-01-20

**Authors:** Christian Pfrepper, Elisabeth Koch, Maria Weise, Roland Siegemund, Annelie Siegemund, Sirak Petros, Michael Metze

**Affiliations:** 1 Division of Hemostaseology, University Hospital Leipzig, Leipzig, Germany; 2 Medical ICU, University Hospital Leipzig, Leipzig, Germany; 3 Department of Cardiology, University Hospital Leipzig, Leipzig, Germany

**Keywords:** anti-Xa activity, low molecular weight heparin, obesity, thromboprophylaxis, tinzaparin, weight-adjusted dosing

## Abstract

**Background:**

The optimal dose of tinzaparin for prophylaxis in obese medical patients is not well defined.

**Objectives:**

To evaluate the anti-Xa activity in obese medical patients on tinzaparin prophylaxis adjusted for actual bodyweight.

**Methods:**

Patients with a body mass index of ≥30 kg/m^2^ treated with 50 IU/kg tinzaparin once daily were prospectively included. Anti-Xa and anti-IIa activity; von Willebrand factor antigen and von Willebrand activity; factor VIII activity; D-dimer, prothrombin fragments; and thrombin generation were measured 4 hours after subcutaneous injection between days 1 and 14 after the initiation of tinzaparin prophylaxis.

**Results:**

We included 121 plasma samples from 66 patients (48.5% women), with a median weight of 125 kg (range, 82-300 kg) and a median body mass index of 41.9 kg/m^2^ (range, 30.1-88.6 kg/m^2^). The target anti-Xa activity of 0.2 to 0.4 IU/mL was achieved in 80 plasma samples (66.1%); 39 samples (32.2%) were below and 2 samples (1.7%) above the target range. The median anti-Xa activity was 0.25 IU/mL (IQR, 0.19-0.31 IU/mL), 0.23 IU/mL (IQR, 0.17-0.28 IU/mL), and 0.21 IU/mL (IQR, 0.17-0.25 IU/mL) on days 1 to 3, days 4 to 6, and days 7 to 14, respectively. The anti-Xa activity did not differ among the weight groups (*P* = .19). Injection into the upper arm compared to the abdomen resulted in a lower endogenous thrombin potential, a lower peak thrombin, and a trend to a higher anti-Xa activity.

**Conclusion:**

Dosing of tinzaparin adjusted for actual bodyweight in obese patients achieved anti-Xa activity in the target range for most patients, without accumulation or overdosing. In addition, there is a significant difference in thrombin generation depending on the injection site.

## Essentials

• The optimal prophylactic dose of tinzaparin in obese medical patients is not well defined.
• Plasma samples from 66 obese patients on tinzaparin (50 IU/kg) were prospectively analyzed.
• The target anti-Xa activity was achieved in 66%, whereas 32% were below the target range.
• Anti-Xa activity did not differ among weight groups and showed no accumulation for over 14 days.

## 1 Introduction

The prevalence of obesity (body mass index [BMI] ≥ 30 kg/m^2^) is rapidly increasing worldwide [1]. About 18% of all adults in Germany already belong to this vulnerable group [2]. Obesity is associated with a chronic inflammation and oxidative stress impairing the protective endothelial function. As a result, obese subjects have higher levels of procoagulant tissue factor, factor (F)VIII, and von Willebrand factor (VWF) as well as an impaired fibrinolysis and a higher platelet activity [3]. Consequently, patients with obesity have a significantly higher risk of venous thromboembolism (VTE) [4]. Therefore, sufficient thromboprophylaxis is particularly important in this patient cohort because adequate thromboprophylaxis is associated with a nearly 90% lower risk of VTE complications [5].

Patients with obesity show differences in the absorption, distribution, metabolism, and elimination of drugs. Barrett et al. found that the clearance of tinzaparin decreases by approximately 25% in obese patients [6]. In addition, absorption may be prolonged after a s.c. injection, which was shown by Sanderink et al. with enoxaparin in obese volunteers [7]. The volume of distribution of low molecular weight heparins (LMWHs) is mainly intravascular, which can be overestimated with a bodyweight–adapted dosage and may lead to an accumulation in obese patients [8]. However, whether a high fixed dose or a bodyweight–adapted dosage is more appropriate in patients with obesity is still a matter of debate. Most studies evaluating thromboprophylaxis with LMWHs in patients with obesity have been carried out with enoxaparin [[9], [10], [11], [12], [13]]. Tinzaparin has different pharmacologic properties compared to enoxaparin and other LMWHs. Tinzaparin has the lowest anti-Xa/anti-IIa activity ratio of all LMWHs and shows the highest inhibition of thrombin generation (TG) at similar anti-Xa activity compared to other LMWHs [14]. Because tinzaparin has a higher molecular weight, the proportion of nonrenal elimination is greater than that of other LMWHs [15]. Therefore, tinzaparin does not lead to a significant accumulation of the anti-FXa activity in elderly patients with impaired renal function [[16], [17], [18]].

In a pharmacokinetic study in healthy obese volunteers, tinzaparin at a prophylactic dose of 75 IU/kg and a therapeutic dose of 175 IU/kg per actual bodyweight resulted in a comparable anti-Xa activity as in nonobese volunteers [19]. A recent case series examined obese medical patients receiving 175 IU/kg tinzaparin per actual bodyweight. Only 13.3% of them had anti-Xa activities below the target range and no overdosing occurred up to a bodyweight of 222 kg [20]. However, there are very few studies focusing on thromboprophylaxis with tinzaparin in obese patients and most of them have been carried out in patients undergoing bariatric surgery, [[21], [22], [23]] whereas there are no published reports on thromboprophylaxis with tinzaparin in obese medical patients. This study aimed to evaluate whether the dosage of tinzaparin per actual bodyweight may lead to an accumulation in obese medical patients and how it affects coagulation.

## 2 Methods

### 2.1 Study design and participants

This open-label prospective study included obese medical patients from September 2018 to March 2021 hospitalized at the University Hospital Leipzig, Germany. Inclusion criteria were a BMI of >30 kg/m^2^ and the administration of tinzaparin per actual bodyweight for thromboprophylaxis as per the institutional protocol of the University Hospital Leipzig. The protocol is based on the administration of >50 IU/kg of current bodyweight, with all patients weighing >200 kg receiving a dose of 12,000 IU. The dosage was based on the prescribing information for tinzaparin that recommends a dose of 50 anti-Xa IU per kilogram of bodyweight in obese patients, [24,25] The recommended way of application was in the upper arm to achieve good absorption and comparability of results. The tinzaparin doses per bodyweight are listed in Table 1.

**Table 1:** Dosage of tinzaparin and distribution of patients and samples among weight groups.

Weight (kg)	≤100	101-119	120-159	160-199	≥200
Dosage (IU)	4500	6000	8000	10,000	12,000
Patients (*n*, %)	18 (27.2)	11 (16.7)	20 (30.3)	6 (9.1)	11 (16.7)
Samples (*n*, %)	29 (24)	21 (17.4)	42 (34.7)	13 (10.7)	16 (13.2)

The exclusion criteria were as follows: age < 18 years, no informed consent, surgery within 14 days prior to inclusion, active malignancy, glomerular filtration rate (GFR) < 20 mL/min, liver cirrhosis child B or C, current pregnancy and lactation, mechanical heart valve, allergy to tinzaparin, or a history of heparin-induced thrombocytopenia.

Blood samples were drawn between day 1 and day 14 after initiation of tinzaparin prophylaxis 4 hours after the s.c. administration of tinzaparin to measure anti-Xa activity; anti-IIa activity; FVIII, VWF antigen, and VWF activity; prothrombin fragment and D-dimer activity; and TG. The following variables were collected from patient records: age, sex, weight, height, diagnosis, and comorbidities. In addition, standard laboratory results including hematology, renal, and liver parameters were taken from the clinical routine. Bleeding events were defined according to the International Society on Thrombosis and Haemostasis nomenclature [26,27].

### 2.2 Blood sampling and laboratory measurement

Blood samples were collected into 3 mL tubes (Sarstedt) containing 3.2% citrate. The samples were processed within 1 hour after the blood collection, centrifuged at 2000 *g* for 20 minutes to prepare platelet-poor plasma, aliquoted, and immediately stored at −80 °C. The aliquots were thawed directly before the analysis. Anti-Xa activity was determined with a chromogenic substrate and a bovine FXa (Hyphen BioMed), and a chromogenic anti-IIa assay with human thrombin (Hyphen BioMed) was used to measure anti-IIa activity. The FVIII activity was assessed by a one-stage clotting test (Siemens Healthineers). In addition, activities of D-dimer, prothrombin fragments, VWF antigen, and VWF (all purchased from Siemens Healthineers) were measured on a BCS analyzer (Siemens Healthineers). The TG assay was carried out on a Fluoroscan Ascent (Fisher Scientific) at a 390/460-nm wavelength using a calibrated automated thrombogram (Diagnostica Stago) activated with 5-pM tissue factor.

### 2.3 Statistical analysis

Descriptive statistics are reported for quantitative variables as either mean ± SD for normally distributed data or as median with IQR otherwise. Qualitative data are given as numbers (percentages). Intergroup comparisons were performed using either t-test, if the data are normally distributed, or the Mann–Whitney U-test or Kruskal–Wallis test otherwise, and all plasma samples from all patients were analyzed. Only the first plasma sample from every group was taken into account for the comparison of time-dependent variables and Friedman’s test was used. Adjustment for multiple testing was performed using the Bonferroni correction. Spearman-Rho correlation coefficients were calculated to assess the effects between the different laboratory parameters and bodyweight. A *P* value of <.05 was considered statistically significant. Statistical analysis was conducted using the program SPSS version 27 (SPSS Inc) and GraphPad Prism version 9.4.0 (GraphPad Software).

### 2.4 Ethical considerations

The study was approved by the Ethics Committee of the Medical Faculty at the University of Leipzig (reference, 182/18-ek) and conducted in accordance with the Declaration of Helsinki. All patients provided written informed consent prior to the inclusion into the study.

## 3 Results

### 3.1 Demographics

A total of 121 plasma samples from 66 white European patients were collected. The median bodyweight was 125 kg (range, 82.2-300 kg) and the median BMI is 41.9 kg/m^2^ (range, 30.1-88.6 kg/m^2^). The majority of the patients (66.7%) received no additional antithrombotics. Indication for platelet inhibitors included coronary heart disease in 10 patients, a history of stroke in 13 patients, and peripheral artery disease in 3 patients. The combination of clopidogrel with anagrelide was given in a patient with essential thrombocythemia, acetylsalicylic acid intolerance, and a history of stroke. Baseline characteristics are listed in Table 2.

**Table 2:** Baseline characteristics.

Female gender	*n* (%)	32	48.5%
Age	Median (range)	60	23-85
Weight (kg)		125	82-300
BMI (kg/m^2^)		41.9	30.1-88.6
GFR (mL/min)		75	21-149
Comorbidities			
Hypertension	*n* (%)	53	80.3%
Diabetes mellitus		31	47.0%
Coronary heart disease		10	15.2%
Chronic heart failure		8	12.1%
Peripheral artery disease		3	4.5%
Chronic venous insufficiency		4	6.1%
Chronic renal failure		15	22.7%
History of venous thromboembolism		2	3.0%
History of stroke		13	19.7%
Additional antithrombotics			
None	*n* (%)	44	66.7%
ASA		18	27.3%
Clopidogrel		1	1.5%
ASA + clopidogrel		2	3.0%
Clopidogrel + anagrelide		1	1.5%

ASA, acetylsalicylic acid; BMI, body mass index; GFR, glomerular filtration rate.

Patients were classified into 5 weight groups according to the tinzaparin dose administered, as shown in Table 1. Plasma samples were divided into 3 groups according to the day after initiation of tinzaparin. On days 1 to 3, days 4 to 6, and days 7 to 14, 45 plasma samples from 41 patients, 40 plasma samples from 37 patients, and 26 plasma samples from 26 patients were available, respectively.

### 3.2 Anti-Xa activity

Median anti-Xa activity was 0.23 IU/mL (0.17-0.29 IU/mL). The target range of the anti-Xa activity of 0.2 to 0.4 IU/mL was achieved in 80 plasma samples (66.1%), whereas 39 (32.2%) plasma samples were below and 2 (1.7%) were above the target range. Bodyweight, BMI, tinzaparin dose, FVIII activity, prothrombin fragments, VWF antigen, and VWF activity were not different in the samples within the target range compared to those <0.2 IU/mL. Patients with an anti-Xa activity below the target range had a higher median endogenous thrombin potential (900 [424-2110] vs 323 [157-1133] nmol/L×min, *P* = .003), a higher peak thrombin (78 [30-254] vs 22 [8-102] nM, *P* = .003), and a lower time to peak (13 [8-18] vs 18 [11-27] minutes, *P* = .01) than those within the target range. In addition, patients below the target range had a higher median D-dimer than those within the target (1.68 [0.92-3.37] vs 1.09 [0.64-1.94] mg/L, *P* = .05).

There was no significant difference in the distribution of the anti-Xa activity among the 5 weight groups (*P* = .19) when plasma samples taken from all days were considered together (part A of Figure).

**Figure:**
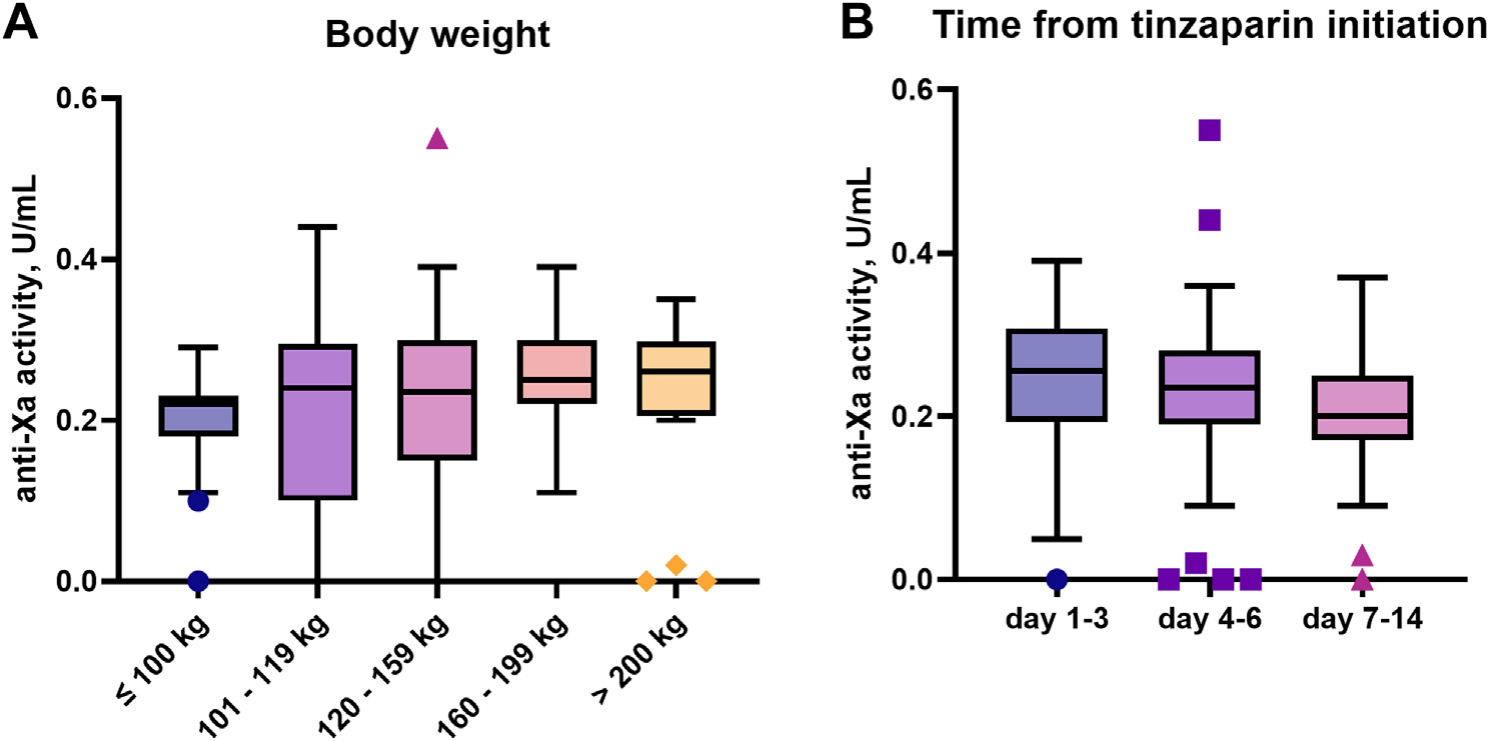
Distribution of the anti-Xa activity in plasma samples from patients with (A) different body weight and (B) at different time points after initiation of tinzaparin.

No accumulation of the anti-Xa activity occurred between days 1 to 3, days 4 to 6, and days 7 to 14 (*P* = .42; part B of Figure).

The anti-Xa activity and the achievement of the anti-Xa target range among the different weight groups are summarized in Table 3.

**Table 3:** Anti-Xa activity and target range among weight groups and day of tinzaparin application

		Days 1-3			Days 4-6			Days 7-14		
Weight group (kg)	Tinzaparin dose (IU)	Samples, *n*	Anti-Xa activity, IU/mL, median [IQR]	Target range, *n* (%)	Samples, *n*	Anti-Xa activity, IU/mL, median [IQR]	Target range, *n* (%)	Samples, *n*	Anti-Xa activity, IU/mL, median [IQR]	Target range, *n* (%)
≤100	4500	3	0.22 [0.21-0.23]	2 (66.7)	10	0.22 [0.17-0.22]	9 (69.2)	10	0.21 [0.17-0.23]	7 (70.0)
101-119	6000	10	0.27 [0.10-0.32]	7 (70.0)	6	0.28 [0.22-0.32]	4 (66.7)	3	0.18 [0.14-0.20]	1 (33.3)
120-159	8000	14	0.24 [0.09-0.32]	8 (57.1)	13	0.25 [0.14-0.31]	7 (53.8)	8	0.20 [0.17-0.30]	4 (50.0)
160-199	10,000	6	0.26 [0.19-0.32]	5 (83.3)	4	0.25 [0.21-0.28]	3 (75.0)	2	0.23; 0.37	2 (100)
≥200	12,000	8	0.27 [0.23-0.31]	8 (100)	4	0.15 [0.01-0.33]	2 (50.0)	3	0.20 [0.10-0.23]	2 (66.7)
All weight groups		41	0.25 [0.19-0.31]	30 (73.2)	37	0.23 [0.17-0.28]	23 (62.2)	26	0.21 [0.17-0.25]	16 (61.5)

### 3.3 Anti-IIa activity

There was a significant difference in the distribution of the anti-IIa activity across the 5 bodyweight groups (*P* = .03) with a significant difference only between patients with 160-199 kg bodyweight (median, 0.055 IU/mL [0.033-0.093 IU/mL]) and those with 101-119 kg bodyweight (median, 0.005 IU/mL [0.00-0.038 IU/mL]). No accumulation of the anti-IIa activity occurred between days 1 to 3, days 4 to 6, and days 7 to 14 (*P* = .14; Supplementary Table 1).

### 3.4 The influence of body mass index and bodyweight on other coagulation parameters

There was a significant, but weak correlation between BMI and D-dimer (r = 0.230, *P* = .01), anti-IIa activity (r = 0.208, *P* = .02), anti-Xa activity (r = 0.196, *P* = .03), and VWF-antigen activity (r = 0.225, *P* = .01). Patients with a BMI of >50 kg/m^2^ (*n* = 39) constituted the highest proportion of patients in the target range (79.5%) followed by patients with a BMI of <40 kg/m^2^ (*n* = 45, 68.9%) and a BMI of 40 to 50 kg/m^2^ (*n* = 37, 48.6%; *P* = .05). There were only 2 samples with an anti-Xa activity of >0.4 IU/mL: one in the group with the BMI of 40 to 50 kg/m^2^ and the other in the group with a BMI of >50 kg/m^2^.

The lowest D-dimer activity was found in patients with a BMI of <40 kg/m^2^, followed by those with a BMI of >50 kg/m^2^ (*P* = .09) and those with a BMI of 40 to 50 kg/m^2^ (*P* = .03). There was no change in D-dimer activity between days 1 to 3, days 4 to 6, and day 7 to 15 (*P* = .74). The anti-IIa activity was not different between these 3 BMI groups (*P* = .06). There was a significant difference in the distribution of the VWF antigen over the 3 BMI groups, with highest values in patients with a BMI of 40 to 50 kg/m^2^ (*P* = .02). Apart from that, no differences in the activity of VWF, FVIII, and prothrombin fragments were found between the BMI groups. The distribution of coagulation parameters among different BMI groups is summarized in Table 4.

**Table 4:** Distribution of coagulation parameters among different BMI groups.

		BMI = 30-40 kg/m^2^ (*n* = 45)	BMI = 40-50 kg/m^2^ (*n* = 37)	BMI > 50 kg/m^2^ (*n* = 39)	*P* value
Anti-Xa activity (IU/mL)	Median [IQR]	0.22 [0.17-0.27]	0.20 [0.13-0.31]	0.26 [0.21-0.30]	.05
Target anti-Xa activity	*n* (%)	31 (68.9)	18 (48.6)	31 (79.5)	.05
Anti-IIa activity (IU/mL)	Median [IQR]	0.02 [0.000-0.045]	0.02 [0.000-0.063]	0.04 [0.005-0.065]	.06
D-dimer (mg/L)		0.92 [0.56-1.79]	1.66 [0.89-2.49]	1.13 [0.70-3.03]	.02
Prothrombin fragments (pmol/L)		241 [178-387]	361 [185-801]	316 [190-659]	.13
VWF antigen (%)		231 [173-281]	336 [209-396]	255 [196-328]	.03
VWF activity (%)		264 [208-300]	261 [216-345]	262 [207-326]	.44
FVIII (%)		168 [134-211]	190 [161-260]	184 [144-221]	.06

BMI, body mass index; F, factor; VWF, von Willebrand factor.

There was a significant difference in the distribution of the D-dimer among the weight groups (*P* = .03). D-dimer were significantly higher in patients weighing >200 kg than those with a bodyweight of 101 to 119 kg (*P* = .03). VWF antigen was significantly lower in the group with ≤100 kg than in the group with 120 to 159 kg (*P* = .01) or in the group with ≥200 kg (*P* = .006). Otherwise, no significant differences in prothrombin fragment, anti-Xa, FVIII, and VWF activity or TG parameters were found between the bodyweight groups. The distribution of laboratory parameters among bodyweight groups is summarized in Table 5.

**Table 5:** Distribution of laboratory parameters among bodyweight groups.

		≤100 kg	101-119 kg	120-159 kg	160-199 kg	≥200 kg	*P* value
Anti-Xa activity (IU/mL)	Median [IQR]	0.22 [0.18-0.23]	0.24 [0.10-0.30]	0.24 [0.15-0.30]	0.25 [0.22-0.30]	0.26 [0.21-0.30]	.19
Target anti-Xa activity	*n* (%)	20 (69)	12 (57.1)	24 (57.1)	11 (84.6)	13 (81.3)	.48
Anti-IIa activity (IU/mL)	Median [IQR]	0.025 [0.00-0.043]	0.005 [0.00-0.038]	0.023 [0.00-0.071]	0.055 [0.033-0.093]	0.033 [0.00-0.044]	.03
D-dimer (mg/L)		1.03 [0.54-2.75]	0.87 [0.63-1.62]	1.49 [0.68-2.41]	1.65 [0.70-2.98]	1.69 [1.02-7.35]	.03
VWF antigen (%)		189 [146-259]	258 [207-356]	290 [195-394]	225 [187-345]	319 [236-370]	.004
VWF activity (%)		252 [178-276]	268 [210-342]	254 [209-321]	279 [212-346]	295 [248-477]	.10
FVIII (%)		163 [131-199]	189 [145-234]	183 [153-253]	179 [143-205]	220 [148-251]	.11

F, factor; VWF, von Willebrand factor.

All patients with a bodyweight of >200 kg received 12,000 IU/mL of tinzaparin. Four patients had a bodyweight of >240 kg (270, 272, 275, and 300 kg) and therefore received <50 IU/kg tinzaparin. Only 1 of these patients had an anti-Xa activity of ˂0.2 IU/mL (0.02 IU/mL), whereas the other 3 patients were within the target range. The median anti-Xa activity of these patients was 0.22 IU/mL (0.07-0.25 IU/mL), with no difference when compared to the rest of the population. However, there were significantly lower anti-IIa activities in these very obese patients compared to the rest of the cohort (*n* = 117, median anti-IIa activity of 0.00 IU/mL [0.00-0.004 IU/mL] vs 0.025 IU/mL [0.000-0.055 IU/mL], *P* = 0.03), and a trend to a higher median endogenous *thrombin* potential (ETP; 1499 nM∗minute [939-2566 nM∗minute] vs 513.8 nM∗minute [204-1478 nM∗minute], *P* = .06).

### 3.5 Correlation of anti-Xa and anti-IIa activity with TG parameters

There was a moderate but significant correlation between the factor anti-IIa and anti-Xa activity with a correlation coefficient of 0.692 (*P* < .001). TG parameters showed a higher correlation with the anti-II activity than with the anti-Xa activity. The highest correlation was found for the anti-IIa activity with the ETP (r = −0.665, *P* < .001) and thrombin peak (r = −0.642, *P* < .001). Correlations between the anti-Xa and the anti-IIa activities with TG parameters are shown in the Supplementary Table 2.

### 3.6 The influence of the injection site on the laboratory results

Tinzaparin was injected into the upper arm in 102 plasma samples (84.3%), whereas 2 injections were administered into the thigh and 17 into the abdominal wall. We found a significant difference in the distribution of all of the 6 TG parameters over the 2 groups. As shown in Table 6, there were a significantly lower ETP, a significantly lower peak thrombin, and a trend to a higher anti-Xa activity in plasma samples taken after the injection of tinzaparin into the upper arm compared to the abdominal wall, but bodyweight and BMI were not different. The differences between the injection into the upper arm and that into the abdomen are summarized in Table 6.

**Table 6:** Comparison of the anti-Xa and IIa activity and thrombin generation parameters at different injection sites.

	Upper arm, *n* = 102		Abdominal wall, *n* = 17		*P* value
	Median	IQR	Median	IQR	
Anti-Xa activity (IU/mL)	0.23	0.19-0.28	0.20	0.08-0.28	.08
Anti-IIa activity (IU/mL)	0.025	0.000-0.056	0.005	0.000-0.048	.14
Lag time (min)	7.8	5.5-11.7	4.7	3.1-8.0	.006
Time to peak (min)	16.0	11.3-26.7	9.6	7.6-14.3	.004
ETP (nM∗min)	455	184-1301	1533	820-2065	.008
Peak thrombin (nM)	32	9.0-134	149	67-249	.008
Velocity index (nM/min)	6.3	0.6-32.0	30.8	12.0-58.0	.02

ETP, endogenous thrombin potential.

### 3.7 The influence of the glomerular filtration rate on the laboratory results

There was no significant difference in the anti-Xa activity (*P* = .81) or the anti-IIa activity (*P* = .18) between patients with a GFR of ≤50 mL/min (*n* = 30) and a GFR of >50 mL/min (*n* = 82). Nevertheless, the mean GFR was significantly higher in patients with an anti-Xa of <0.2 IU/mL than in those with an anti-Xa activity within the target range (80.5 [SD ± 29.4] vs 68.5 [SD ± 25.4], *P* = .03). Patients with a GFR of >50 had significantly lower prothrombin fragment levels (277 pmol/L [IQR, 168-446 pmol/L] vs 387 pmol/L [IQR, 228-830 pmol/L], *P* = .01), a lower FVIII activity of 170 mg/L (IQR, 139-208 mg/L) vs 207 mg/L (IQR, 163-255 mg/L), *P* = .03, and lower D-dimer activity (1.03 mg/L [IQR, 0.63-1.92 mg/L] vs 1.70 mg/L [IQR, 0.86-3.17 mg/L], *P* = .03).

### 3.8 Clinical outcome

There were no major or clinically relevant nonmajor bleeding events during tinzaparin prophylaxis. No thrombotic complications occurred during hospitalization, and there was no readmission due to a thrombotic event.

## 4 Discussion

This study shows that thromboprophylaxis with tinzaparin per actual bodyweight does not lead to an overdose or accumulation in obese medical patients. In addition, the majority of the patients (66.1%) achieved an anti-Xa activity in the target range of 0.2 to 0.4 IU/mL, with only very few patients above the target range. This shows that even high absolute doses of tinzaparin for thromboprophylaxis in obese medical patients appear to be safe.

Only limited data exist on tinzaparin prophylaxis in obese patients. The first pharmacodynamic study focusing on tinzaparin pharmacology in obesity included healthy subjects with up to a bodyweight of 165 kg. Patients administered with a single tinzaparin dose of 75 U/kg of actual bodyweight showed similar anti-Xa activities compared to subjects of normal weight [19,28]. We found that no accumulation occurred after repeated dosing for more than 7 days. This is consistent with the study by Mahe et al. [18] in patients with renal failure, which showed no accumulation of anti-Xa activity by tinzaparin after 8 days. Tseng et al. [23] investigated the safety of weight-adjusted prophylaxis with 75 IU/kg tinzaparin after bariatric surgery and concluded that it appears to be a safe strategy with few major bleeding events (1.6%) and a low rate of venous thromboembolism (0.5%) within 30 days after bariatric surgery. Unfortunately, the anti-Xa activity was not monitored in that study. Most data exist only on the use of enoxaparin for thromboprophylaxis in bariatric surgery. These studies conclude that patients receiving a weight-based enoxaparin prophylaxis are more likely to have their anti-Xa activity in the target range than with a fixed-dose regimen, whereas thrombotic complications are rare [[29], [30], [31], [32], [33], [34]].

Nevertheless, whether the anti-Xa activity correlates with the clinical outcome, particularly venous thromboembolism or bleeding, is still a matter of debate [35]. Bara et al. [36] examined 440 patients who underwent total hip replacement surgery and found no correlation between the anti-IIa or anti-Xa activity and the clinical outcome. Cooper et al. reported that not weight-adjusted tinzaparin dosing but rather the addition of acetylsalicylic acid results in persistent wound drainage after knee and hip arthroplasty [37]. In a recently published study on patients with gynecologic cancer receiving prophylactic treatment with tinzaparin, the anti-Xa activity was significantly lower in patients who developed VTE than in those without a thrombotic event, and the anti-Xa activity was significantly higher in patients receiving a weight-adjusted prophylaxis compared to a fixed dose of 4500 U of tinzaparin [38]. According to these findings, some current guidelines and expert opinion suggest a bodyweight-adjusted VTE prophylaxis in obese patients [[39], [40], [41]]. However, most international guidelines do not address dosing in patients with obesity because of the lack of evidence [[42], [43], [44], [45], [46], [47]]. For high-risk bariatric surgery patients, the measurement of the anti-Xa activity should be considered, [48] as there is a 28-fold increase in the mortality risk once venous thromboembolism occurs postoperatively [49]. As the target range of anti-Xa was only achieved in two-thirds of the patients, we suggest that its measurement is warranted especially in the context of high mortality with thromboembolism.

The absorption of LMWH in obese patients has not been fully understood. We found a trend to a higher anti-Xa activity after the administration of tinzaparin into the upper arm compared to that into the abdominal wall. In addition, injection into the abdominal wall led to significantly lower ETP and peak thrombin values. This might be clinically associated with less effective thromboprophylaxis. However, this observation might be biased by the uneven sample distribution (abdominal wall: *n* = 17; upper arm: *n* = 102), so further confirmation studies are needed. In addition, the difference could be caused by delayed absorption after injection into the abdominal wall because of lower venous return, [50] so that the plasma peak occurs 4 hours after injection. Local skin movement is limited in the abdomen compared to the upper arm region, which impacts s.c. blood flow and absorption [51]. Studies focusing on the differences between injection sites, especially in obese people, are very limited. Sanderink et al. [7] examined the pharmacokinetic parameters on days 1 and 4 after daily s.c. enoxaparin administration in 24 nonobese and obese volunteers. They found that after s.c. injection at day 4, the absorption time is prolonged in obese volunteers by 1 hour (*P* = .005), although the maximum anti-Xa activities are similar in both groups. In contrast, in the pharmacokinetic study of tinzaparin reported by Hainer et al. [19], peak anti-Xa activity in the cohort of obese healthy volunteers did not differ from that of the normal-weight control group. Despite the limited evidence, we suggest administration of tinzaparin in the upper arm in obese patients to achieve better efficacy of the thromboprophylaxis and more reliable measurements of anti-Xa activity after 4 hours.

Obesity seems to be associated with a higher risk of VTE [52]. This obesity-induced prothrombotic state results from the interplay of physical immobility and proinflammatory and hypofibrinolytic effects [53]. Adipocytes secrete inflammatory cytokines that stimulate the endothelium and platelets, leading to increased tissue factor expression and TG [54,55] and an impaired fibrinolysis [3]. TG is known to return to lower values after weight-loss following bariatric surgery [56]. As C-reactive protein decreases too, fewer proinflammatory stimuli are a possible explanation [57]. With regard to FVIII and VWF, we confirmed that BMI seems to be associated with higher VWF and FVIII levels as demonstrated by Atiq et al [58]. Finally, D-dimer—likely inflammatory-driven—were elevated in our patient cohort. This confirms the data of Franco et al., [59] who reported elevated D-dimer in obese patients in correlation to the waist-to-hip ratio. Elevated D-dimer have been reported to predict VTE in acutely ill medical patients [[60], [61], [62]]. In the MEDENOX trial that included nonobese patients, 40-mg of enoxaparin but not 20-mg of enoxaparin or placebo resulted in a small but significant reduction in the elevated baseline D-dimer activity after 10 days [62]. VTE was documented only among patients with elevated D-dimer at baseline that persisted after 10 days. In contrast, D-dimer did not decrease over time in our cohort of obese medical patients despite a weight-adapted thromboprophylaxis. This might be explained by the proinflammatory status associated with obesity. Considering that one-third of our patients did not achieve the targeted anti-Xa activity, this might justify an even higher dose of thromboprophylaxis. However, our cohort was too small to draw definite conclusions and was not powered for endpoints such as VTE occurrence.

With regard to TG, we found higher correlations between the TG parameters and the anti-IIa compared with the anti-Xa activity. The fact that TG measurements are more influenced by the anti-IIa activity than by the anti-Xa activity of LMWH has been described previously [14,63]. As a result, tinzaparin has a higher effect on TG than enoxaparin, nadroparin, or dalteparin because of the lower anti-Xa/anti-IIa ratio. However, whether this effect is clinically relevant or just an effect of the more downstream inhibition of TG by the anti-IIa activity or whether a low anti-IIa activity may indicate a less efficient prophylaxis sooner remains unclear. Therefore, our data cannot be generalized to other LMWHs.

## Limitations

This study has several limitations because of the relatively small cohort of patients and the uneven distribution among the different bodyweight and BMI groups. Therefore, despite that correction for multiple testing was made, our results should be interpreted with caution and confirmed in a larger cohort. In addition, the observation period was short, and no follow-up regarding thrombotic complications and no duplex sonography were performed to exclude occult thrombosis. Therefore, no final conclusion can be drawn regarding the effectiveness of the dosing regimen. Nevertheless, this is, to our knowledge, the first study that examined the bodyweight-adjusted dosing of tinzaparin in obese medical patients by collecting anti-Xa and anti-IIa peak values.

## Summary

Our data suggest that bodyweight-adjusted dosing of tinzaparin results in an effective thromboprophylaxis in obese medical patients, with the majority of patients achieving the anti-Xa target range. No accumulation of the anti-Xa activity occurred over a period of 14 days. Furthermore, we found evidence that s.c. injection into the abdominal wall may lead to a less effective inhibition of TG than the administration into the upper arm. Therefore, we recommend administration of weight-adjusted tinzaparin into the upper arm in patients with obesity. Nevertheless, further research is required to prove our findings.
