# Supplementary material for: Weight-adjusted dosing of tinzaparin for thromboprophylaxis in obese medical patients
Source: Res Pract Thromb Haemost. 2023 Jan 20;7(2):100054. doi: 10.1016/j.rpth.2023.100054 (PMC9975291; doi:10.1016/j.rpth.2023.100054)
Supplement: Supplementary Material [file mmc1.docx]

**Supplementary material**

Supplementary table 1: Anti-IIa activity over weight groups and day of administration

|  |  | Day 1 - 3 | | Day 4 - 6 | | Day 7 - 14 | |
| --- | --- | --- | --- | --- | --- | --- | --- |
| Weight group (kg) | Tinzaparin dose (IU) | Samples, n | Anti-IIa activity, IU/mL, median [IQR] | Samples, n | Anti-IIa activity, IU/mL, median [IQR] | Samples, n | Anti-IIa activity, IU/mL, median [IQR] |
| ≤ 100 | 4500 | 3 | 0.025  [0.013 – 0.035] | 10 | 0.028  [0.004 – 0.046] | 10 | 0.023  [0.000 – 0.031] |
| 101 – 119 | 6000 | 10 | 0.005  [0.000 – 0.059] | 6 | 0.005  [0.000 – 0.071] | 3 | 0.025  [0.013 – 0.025] |
| 120 – 159 | 8000 | 14 | 0.005  [0.000 – 0.074] | 13 | 0.020  [0.000 – 0.078] | 8 | 0.033  [0.006 – 0.091] |
| 160 – 199 | 10000 | 6 | 0.058  [0.011 – 0.091] | 4 | 0.050  [0.034 – 0.063] | 2 | 0.035; 0.160 |
| ≥ 200 | 12000 | 8 | 0.020  [0.000 – 0.050] | 4 | 0.023  [0.001 – 0.078] | 3 | 0.030  [0.015 – 0.038] |
| All patients |  | 41 | 0.001  [0.000 – 0.065] | 37 | 0.030  [0.000 – 0.055] | 26 | 0.025  [0.004 – 0.041] |

Abbreviation: kg, kilogram; IU, international units; IQR, interquartile range.

Supplementary table 2: Correlations of anti-Xa and anti-IIa activity with thrombin generation

|  |  | **correlation coeffizients (r)** | **significance (p)** |
| --- | --- | --- | --- |
| anti-IIa activity | anti-Xa activity | 0.692 | < 0.001 |
| ETP | a-IIa | - 0.665 | < 0.001 |
|  | a-Xa | - 0.394 | < 0.001 |
| Peak thrombin | a-IIa | - 0.642 | < 0.001 |
|  | a-Xa | - 0.400 | < 0.001 |
| Velocity index | a-IIa | - 0.563 | < 0.001 |
|  | a-Xa | - 0.398 | < 0.001 |
| Lag time | a-IIa | 0.455 | < 0.001 |
|  | a-Xa | 0.241 | 0.023 |
| Time to peak | a-IIa | 0.598 | < 0.001 |
|  | a-Xa | 0.374 | < 0.001 |

Abbreviation: ETP, endogenous thrombin potential; a-IIa, anti-IIa activity; a-Xa, anti-Xa activity.
